# High-frequency measured turbidity as a surrogate for phosphorus in boreal zone rivers: appropriate options and critical situations

**DOI:** 10.1007/s10661-020-08335-w

**Published:** 2020-05-15

**Authors:** Maria Kämäri, Marjo Tarvainen, Niina Kotamäki, Sirkka Tattari

**Affiliations:** 1 Finnish Environment Institute, Latokartanonkaari 11, FI-00790 Helsinki, Finland; 2 Centre for Economic Development, Transport and the Environment for Southwest Finland, P.O. Box 236, FI-20101 Turku, Finland; 3 Finnish Environment Institute, Survontie 9A, FI-40500 Jyväskylä, Finland

**Keywords:** River water quality, In situ monitoring, Proxy relations, Local calibration, Linear regression

## Abstract

In situ high-frequency measured turbidity can potentially be used as a surrogate for riverine phosphorus (P) concentrations to better justify the effectiveness of nutrient loss mitigation measures at agricultural sites. We explore the possibilities of using turbidity as a surrogate for total phosphorus (*TP*) and particulate phosphorus (*PP*) in four snowmelt-driven rivers draining agricultural clayey catchments. Our results suggest slightly stronger relationship between in situ measured turbidity and *PP* than between turbidity and *TP*. Overall, linear *TP* and *PP* regressions showed better error statistics in the larger catchments compared with their sub-catchments. Local calibration of the in situ sensors was sensitive to the number of high P concentration discrete water samples. Two optional calibration curves, one with and one without influential data, resulted in a 17% difference in the estimated mean *TP* concentrations of a snowmelt storm contributing 18% of the annual discharge volume. Accordingly, the error related to monthly mean *TP* estimates was the largest in spring months at all sites. The addition of total dissolved phosphorus (*TDP*) improved the model performance, especially for sites where the *TDP/TP* ratio is large and highly variable over time. We demonstrate how long-term discrete samples beyond sensor deployment can be utilized in the evaluation of the applicability range of the local calibration. We recommend analysing the validity of P concentration estimates, especially during high discharge episodes that contribute substantially to annual riverine nutrient fluxes, since the use of surrogates may introduce large differences into the P concentration estimates based on selected local calibration curves.

**Electronic supplementary material:**

The online version of this article (10.1007/s10661-020-08335-w) contains supplementary material, which is available to authorized users.

## Introduction

Phosphorus (P) is one of the main fertilizers used in agriculture. However, excess P losses from agricultural areas to receiving water bodies can cause water quality impairments. It has been shown that field and lake percentages as well as soil type strongly control total phosphorus (*TP*) loss from catchments in Finland (Röman et al. 2018; Vuorenmaa et al. 2002). Thus, TP transported in rivers is often monitored to evaluate excess P losses and the effectiveness of mitigation measures intended to constrain P losses from agricultural sites (e.g., Tattari et al. 2017). *TP* can be divided into two operational fractions: total dissolved phosphorus (*TDP*) and particulate phosphorus (*PP* = *TP* ˗ *TDP*) (Haygarth and Sharpley 2000). *PP* is usually the dominant fraction in field runoff from drained clay soils (Ulén and Persson 1999).

In situ measured turbidity (*TURB*) has been used as a surrogate for *TP* or *PP* in several studies (Grayson et al. 1996; Hopkins et al. 2017; Horsburgh et al. 2010; Lannergård et al. 2019; Spackman Jones et al. 2011; Stubblefield et al. 2007; Valkama and Ruth 2017; Villa et al. 2019). Recent studies have suggested that the river nutrient load is better captured with high-frequency optical sensors than with low-frequency water sampling combined with continuous discharge data (Stutter et al. 2017; Valkama and Ruth 2017). However, *TP* flux estimation associated with storm flow events is highly sensitive to local sensor calibration, i.e., conversion of sensor turbidity values into P concentrations (Lannergård et al. 2019). Thus, site-specific local calibration of the raw data of the optical sensors into representative turbidity units or water quality concentrations is needed depending on the sensor used and is mandatory when surrogates are used. Automatic water sample collection based on flow rate changes or on preset water quality conditions provides a good surrogate relationship according to Minaudo et al. (2017) and Melcher and Horsburgh (2017). However, autosamplers are typically available only at a few monitoring sites, and large flow peaks are often missed by manual sampling, especially if hydrograph slopes are steep. Despite the existence of published guidelines for in situ data processing (e.g., Pellerin et al. 2013), there is a need to define practices regarding how to conduct the efficient sampling needed for local calibration by minimising costs and achieving acceptable accuracy in the resulting water quality and nutrient yield estimates (Horsburgh et al. 2010).

Turbidity is a suitable surrogate for *TP* only if a reasonable relationship between these constituents is found (Villa et al. 2019). The physicochemical properties of suspended sediment impact the capacity of suspended solids to transport *PP*, which is largely site dependent and varies temporally (House and Denison 2002; Van der Perk et al. 2007). Factors such as land management may introduce additional variations to the relationship between *TP* and turbidity. Local assessment of the appropriateness of surrogate relationships between turbidity and *TP* has been stressed in several studies (e.g., Grayson et al. 1996; Ruzycki et al. 2014). Stutter et al. (2017) concluded that the surrogate relationship between *PP* and turbidity was not transferable to a neighbouring catchment with similar soils and land cover. In addition, the particle size distribution of suspended sediments and bedload can be different in low- and high-flow situations (Pfannkuche and Schmidt 2003) and may impact the relationship between turbidity and *TP*. In some sites, there is a positive relationship between *TP* concentration and the specific surface area of suspended sediments (Evans et al. 2004). Thus, for example, the storm-related *TP* concentration in suspended solids can be much higher during the rising limb of the hydrograph compared with the falling limb or baseflow (Evans et al. 2004). Nonlinearity and hysteresis effects were taken into account by Minaudo et al. (2017), who found more reliable *TP* load estimates than those calculated with a linear surrogate relationship between *TP* and turbidity. The seasonal variation in the relationship between *TP* concentration and suspended sediments, as well as differences in P retention mechanisms, between two rivers, was reported by Evans et al. (2004). Differences in the storm-related concentration-discharge responses of *TP* and unfiltered molybdate-reactive P may be explained by the surface and subsurface transport of P fractions and the wetness of the catchment (Outram et al. 2014). Dissolved P loading may originate from scattered sources, such as fields with high soil P levels (Uusitalo et al. 2007), livestock grazing and manure spreading or point sources.

The *TDP*/*TP* ratio may vary interannually and depend on catchment properties (Johnes 2007). Thus, variations in the *TDP*/*TP* ratio may degrade the usability of turbidity as a surrogate for *TP*. Consequently, the uncertainty of *TP* estimates using turbidity as a proxy needs to be better quantified. To improve *TP* and *PP* estimates with a turbidity surrogate, additional surrogate constituents or explanatory variables such as snowmelt, discharge, orthophosphorus, chlorophyll A or chloride have been suggested by Spackman Jones et al. (2011) and Schilling et al. (2017). Haygarth et al. (2005) suggested that with care in calibration, turbidity could potentially act as a surrogate for *PP* in stormflows; thus, we study both *TP*’s and *PP*’s relationship with turbidity.

Often, the constraint in local sensor calibration is that high flow situations are rarely sampled in regular monitoring, especially when the hydrograph slopes are steep. We test a novel and cost-effective practice to utilize the available long-term river water quality monitoring data collected prior to sensor deployment as reference data for local calibration. In optimal situations, this may provide additional confidence in site-specific local sensor calibration. The objectives of the study are as follows:

1. To evaluate the performance of in situ UV-Vis sensors in four river basins to produce turbidity, *TP* and *PP* estimates.
2. To determine how turbidity acts as a surrogate for *PP* and *TP* using long-term laboratory data.
3. To quantify the effect of potentially influential samples on *TP* concentration estimates due to changes in the local calibration equations of the UV-Vis sensors.
4. To test the transferability of a local *TP* calibration equation of a UV-Vis sensor.
5. To evaluate the impact of *TDP* on the surrogate relationship between turbidity and *TP.*

## Study areas

Four rivers from Southwest Finland were selected based on their in situ water quality, data availability and representativeness of the area and size. The basin sizes varied from smaller sub-basins (15–233 km^2^) to larger main basins (727–1317 km^2^) (Table 1). The large rivers of Aurajoki and Eurajoki flow into the Baltic Sea. The Savijoki River is a small tributary of the Aurajoki River, whereas the Yläneenjoki River belongs to the Eurajoki basin and flows into Lake Pyhäjärvi (154 km^2^), which is the source of the Eurajoki River and accounts for 25% of the Eurajoki basin.

**Table 1:** Study area characteristics. Land classification is largely based on the 2012 CORINE land cover classification, and the soil types were obtained from a digital soil map (1:200,000; Lilja et al. 2006). Dominant crop types were obtained from a parcel register owned by the Agency for Rural Affairs

River/basin name	1. Aurajoki	2. Savijoki	3. Eurajoki	4. Yläneenjoki
Soil	Vertic Cambisol, Fibric/Terric Histosol, Dystic Leptosol	Vertic Cambisol, Dystic Leptosol, Lithic Leptosol	Haplic Podzol, Dystric Gleysol, Fibric/Terric Histosol, Eutric Regosol, Eutric Gambisol, Lithic Leptosol	Lithic Leptosol, Fibric/Terric Histosol, Eutric Gambisol, Haplic Podzol
Watershed size (km^2^)	727	15.2	1317	233
Urban and industrial areas (%)	4.5	2.1	4	3.4
Forest (%)	41.2	47.7	52.9	65.4
Peat or wetland (%)	7.8	0.8	2	3.3
Lakes (%)	0.2	0	25	0.1
Field (%)	37	39	16	28
Pasture (% of agricultural fields)	18.1	12	17.2	20
Mean field slope (%)	1.8	2.7	1.2	1.8
Dominant crop types	Spring cereals, grass, autumn cereals	Spring cereals, grass, root crops	Grass, spring cereals, autumn cereals	Spring cereals, grass, autumn cereals
Mean runoff (L s^−1^ km^−2^)	9.5 (1990–2017) 8.2 (2009–2013)	11 (1981–2010) 9.5 (2009–2015)	6.3 (1990–2017) 6.0 (2009–2015)	7.7 (1990–2016) 6.9 (2013–2015)
Median TP (1990–2017) (μg L^˗1^)	160 (*n* = 1160)	110 (*n* = 926)	44 (*n* = 131)	100 (*n* = 869)

Most of the agricultural land of the Aurajoki and Savijoki basins is classified as a Vertic Cambisol (75%), which is a highly erodible soil. Thus, the river water in these two rivers is highly turbid (Fig. A1). Agricultural fields in the Savijoki area are steeper than those in the entire Aurajoki area. These catchments do not include any large lakes. The Aurajoki catchment is representative of watersheds located in Southwest Finland.

The Yläneenjoki and Eurajoki rivers represent chained river-lake systems. In the Yläneenjoki catchment, the share of agricultural land is slightly smaller than that at the Aurajoki site, while in the Eurajoki catchment, the share is even lower (Table 1). In these catchments, the main soil types are Haplic Podzols and Lithic Leptosols and thus different compared with those of the Aurajoki and the Savijoki catchments. The turbidity level is the lowest in the Eurajoki basin. On the coastal plains of western Finland, acid sulphate soils in former sea bed areas are common. Some agricultural land areas in the Eurajoki basin are classified as acid sulphate soils, which are highly productive. Periodically, high acidity and high concentrations of sulphate and metals are associated with stream waters affected by acid sulphate soils (Nyberg et al. 2012). Vuorenmaa et al. (2002) studied small agricultural catchments and found that losses of *TP* were lower from agricultural acid sulphate soils compared with clayey agricultural soils. Thus, the low field percentage and acid sulphate soils in Eurajoki seem to explain the low *TP* and *PP* concentrations there compared with other sites.

In Southwest Finland, the average annual rainfall is 723 mm years^−1^ (1981–2010), and the mean air temperature is 5.5 °C (Pirinen et al. 2012). The median *TP* concentrations (Table 1) indicate poor chemical status for the Aurajoki, Savijoki and Yläneenjoki rivers, whereas the chemical status based on *TP* concentration is good in Eurajoki (Aroviita et al. 2012). However, the ecological status of Eurajoki is moderate based on data from 2012 to 2017. Long-term laboratory data indicate that on average, more than 70% of *TP* is in particulate form in the studied rivers (Fig. A1). Earlier studies provided information on the P loading of the Eurajoki and Yläneenjoki (Koskiaho et al. 2010; Ventelä et al. 2007), Aurajoki (Korppoo et al. 2017) and Savijoki (Granlund et al. 2005; Rekolainen et al. 1991; Tattari et al. 2017) rivers.

## Data and methods

### Discrete water samples and laboratory analyses

The water monitoring interval ranged from subdaily to once-a-month sampling. Routine water quality surveillance was conducted over the period 1990–2017. At Savijoki, an automated water sampler was occasionally applied from 2007 to 2009. At the Eurajoki site, water samples were not collected regularly prior to the in situ sensor installation in 2009; thus, long-term monitoring data were not available from that site.

Standardized methods were used in chemical analyses, and we applied P fraction classification according to Haygarth and Sharpley (2000) (Table A1). *TP* was determined colourimetrically by the molybdenum blue method (Murphy and Riley 1962) using ascorbic acid as a reductant and the digestion of organic P compounds with potassium peroxodisulphate. *TDP* was determined similarly to *TP*, but the samples were filtered first using Nuclepore polycarbonate membranes with a 0.4 μm pore size. *PP* was obtained by subtracting *TDP* from *TP*. The nephelometric method was applied for turbidity laboratory analyses. *TP*, *TDP* and turbidity data were retrieved from the open source database of the Finnish Environment Institute (www.syke.fi/en-US/Open_information). The long-term monitoring data were used to evaluate the feasibility of building linear least square *TP* or *PP* estimation models with turbidity as a surrogate. In addition, the laboratory data were used in the local calibration of the sensors.

### In situ optical turbidity measurements

We used s::can UV-Vis spectrophotometers (s::can Messtechnik GmbH, Austria) to measure light absorbance in the wavelength range from 220 to 720 nm. The manufacturer has defined global algorithms that interpret the absorbance spectrum introduced by turbid matter in water, which enables the spectrophotometer to produce raw turbidity values. In the case of s::can sensors, a site-specific local calibration is needed to convert raw units into representative turbidity values. The sensors were measured at 30-min intervals during the following periods: January 2010 to September 2013 (Aurajoki, Site 1), August 2009 to September 2013 (Savijoki, Site 2), June 2009 to December 2015 (Eurajoki, Site 3) and November 2013 to December 2015 (Yläneenjoki, Site 4). A few short periods existed when sensor data were not retrieved for Eurajoki. The sensor at Aurajoki was removed from the river for the ice clearance periods of spring 2010 and 2011 to avoid damage to the instrument. The optical path lengths of the sensors were either 2 mm (Sites 1 and 4) or 5 mm (Sites 2 and 3). The accuracy of a 2 mm sensor for raw turbidity values is ± 12 units, and for 5 mm sensors, it is ± 3 units, based on measurement experience in Finnish rivers. The optimal turbidity measurement ranges of the 2 mm and 5 mm sensors are 0–1000 FNU and 0–2500 FNU, respectively. The spectrophotometers used simultaneously measure turbidity and nitrate nitrogen. The optical path length of each spectrophotometer is such that the device is capable of measuring both turbidity and nitrate nitrogen as well as possible, taking into account the water quality of the river. All probes are equipped with automatic compressed air cleaning operating every other hour. Manual cleaning was conducted at least fortnightly during the growing season and every 3 weeks during the cold season. Baseline checking for the spectrometers was performed with Milli-Q water once a year. Degradation of the sensor lamps, which were no more than 6 years old, was not detected at baseline.

In quality control, the sensor data were visually inspected to detect data gaps and anomalies. Water and air temperature time series, as well as water level fluctuation, sensor nitrate nitrogen reading, and sensor maintenance notes, were used in the quality control. Sensor data spikes, e.g., due to icing or biological disruption, were detected and removed from the raw data time series. A few outliers were also removed from the laboratory data when the ratio of analysed P to turbidity was abnormal. After outlier removal, we were able to perform the local sensor calibration and match quality-controlled raw sensor turbidity values with discrete laboratory-determined water quality data. The water samples used in the calibration were collected as close as possible to the sensors. The potential time difference between grab sampling and sensor reading was resolved by pairing the discrete samples with the nearest sensor reading in time.

### Statistical analysis

Prior to analysis, two-way relationships between *TP* or *PP* and turbidity were examined. Based on scatterplots, linearity of the relationships between variables was assumed, and simple linear regression models were fitted to the data. The models are in the form *y = αx + β*, where *TP* or *PP* is the response (*y*) variable, turbidity is the explanatory (*x*) variable, and *α* and *β* are the slope and the intercept of the regression line, respectively. Normality, heteroscedasticity and independency of the model residuals were tested and found not to drastically violate the regression model assumptions. This means that the standardized residuals are symmetrically distributed, they are not clustered in any way and they do not show any clear patterns. In general, when the models are used purely for predictions, slight violations of the model assumptions are not considered a significant problem (Kutner et al. 2004).

Several goodness-of-fit statistics were used to evaluate the performance of the models (Table A2). The coefficient of determination (*R*^2^) was used to assess how well the model fits the data or how strong the linear relationship is between values. The model standard percentage error (*MSPE*) and relative percentage difference (*RPD*) were used to assess the linear regression models. *RPD* was calculated between estimated values and laboratory-determined values. *MSPE* and *RPD* can be used to compare separate regression models (Rasmussen et al. 2009) since these standardized statistics are independent of the mean concentration (Hardison 1969). The range of a value plus or minus twice the *RMSE* produces the 95% confidence limits for the prediction of the response variable (Kleinbaum and Kupper 1978). Spearman’s correlation (*r*_*s*_) was used to assess the relationship between laboratory-determined *TP* or *PP* and turbidity. Simple linear regression models between *TP* or *PP* and turbidity were formed using

i) sensor deployment period laboratory data and raw turbidity sensor data (i.e., local calibration)
ii) long-term laboratory data
iii) sensor deployment period laboratory data

In local calibration (i), it is especially important that the sample size is sufficiently large with respect to the measurement range, and the samples represent the whole flow or turbidity range (Pellerin et al. 2013). Using long-term laboratory data (ii), it is possible to determine whether turbidity acts better as a surrogate for *PP* than for *TP*. We hypothesise that the larger amount of long-term data and potentially more representative long-term data would provide more insight into the relationship of the water quality constituents. A comparison between regressions ii and iii is made to explore whether the resulting regressions based on different amounts of data are similar. In that case, the local sensor calibration might be applicable to a wider P concentration range than the discrete samples of the sensor deployment period.

Linear regression models can be sensitive to observations that diverge from the overall pattern in sample data. These so-called influential observations are not erroneous but true extreme values that have an effect on the model slope. This in turn has an influence on the estimates (e.g., the estimates of *TP* and *PP* in this case). We identify visually potentially influential data pairs and use Cook’s distance (*D*_*i*_) (Cook 1977) to quantify their potential influence on linear regression models. We hypothesise that highly influential data pairs are those when *D*_*i*_ is larger than one, as suggested by Cook and Weisberg (1982). Their effect on local calibration regression equations (i) is shown. We also quantify the impact of potentially influential samples on monthly and event-scale mean *TP* concentration estimates produced with in situ sensors. In addition, we use multiple linear regression (SPSS 23.0) to test the effect of adding the *TDP* concentration as an additional explanatory variable to the local *TP* calibration equations.

## Results

### The quality and applicability of *TP* and *PP* estimates of the in situ turbidity sensors

The local *TP*, *PP* and turbidity calibration results are shown in Tables 2 and 3 and in the Supplementary material (Fig. A2). The median *RPD* between turbidity estimates of the sensors and laboratory analysed turbidity varied between 22 and 29% depending on the site considered (Table 3). Hence, there was not a perfect fit between the sensor and laboratory-determined turbidity. Thus, we used the quality-checked sensor raw turbidity and not the calibrated sensor turbidity in the formation of the local *TP* and *PP* calibration equation to avoid the potential additional imprecision between the two different turbidity determination methods.

**Table 2:** Determined regression models were used in local calibration to convert in situ sensor raw turbidity values into calibrated total phosphorus (*TP*), particulate phosphorus (*PP*) and turbidity (*TURB*) estimates. The explanatory variable (*x*) is the raw sensor turbidity. Summary statistics include the number of discrete data pairs (*n*), the maximum Cook’s distance (*Di*), the standard error (*SE*) of the slope, the coefficient of determination (*R*^2^), the root mean square error (*RMSE*), the model standard percentage error (*MSPE*) and the median relative percentage difference (*RPD*)

Site	Regression model	*n*	Max *D*_*i*_	*SE* slope	Laboratory value, mean (μg l^−1^)/(FNU)	Laboratory values, min–max (μg L^˗1^)/ (FNU)	Sensor-calibrated values, min–max (μg L^˗1^)/(FNU)	*R*^2^	*RMSE* (μg L^˗1^)/(FNU)	*MSPE* (%)	Median *RPD* (%)
1 Aurajoki	*TP* = 2.73*x* + 89.34	125	0.55	0.122	162	48–670	89–639	0.80	40.0	25	19
	*PP* = 2.14*x* + 63.12	120	1.01	0.117	117	31–423	68–434	0.74	32.0	27	16
	*TURB* = 2.13*x* + 16.88	123	2.14	0.052	74	12–490	17–446	0.93	17.1	23	16
2 Savijoki	*TP* = 2.29*x* + 89.50	138	3.74	0.107	166	64–1100	94–1200	0.77	64.3	39	26
	*PP* data not available	-	-	-	-	-	-	-	-	-	-
	*TURB* = 2.69*x* − 4.31	138	0.95	0.053	86	4–1300	1–1302	0.95	31.5	37	21
3 Eurajoki	*TP* = 4.18*x* + 12.60	104	9.42	0.210	50	10–300	16–219	0.80	15.3	30	21
	*PP* = 4.01*x* + 3.12	98	9.70	0.194	40	6.5–276	6.3–202	0.82	14.1	35	22
	*TURB* = 1.58*x* − 1.25	104	0.44	0.065	13	2–78	0–77	0.85	4.7	36	29
4 Yläneenjoki	*TP* = 1.57*x* + 116.91	33	557	0.056	175	64–1400	117–1410	0.96	45.1	26	30
	*PP* = 1.56*x* + 70.60	33	687	0.052	129	0–1341	71–1351	0.97	41.7	33	30
	*TURB* = 1.31*x* + 25.15	33	880	0.023	74	7.6–1100	25–1105	0.99	18.4	26	31

**Table 3:** The local calibration equations of the sensors without potentially influential data pairs for total phosphorus (*TP*), particulate phosphorus (*PP*) and turbidity (*TURB*). The explanatory variable (*x*) is the raw sensor turbidity

Site	Regression model	Number of data pairs	Max *D*_*i*_	*SE* slope	Laboratory value, mean (μg l^−1^)/(FNU)	Laboratory values, min–max (μg L^˗1^)/(FNU)	Estimatesmin–max (μg L^˗1^)/(FNU)	*R*^2^	*RMSE* (μg L^˗1^)/(FNU)	*MSPE* (%)	Median *RPD* (%)
1 Aurajoki	^a)^*TP* = 2.60*x* + 92.04	123	0.85	0.158	156	48–360	92–424	0.69	40.0	26	20
	^b)^*TP* = 3.038x + 83.99	121	0.21	0.163	154	48–360	84–357	0.74	35.9	23	18
	^c)^*PP* = 2.297*x* + 60.63	119	0.82	0.108	117	31–423	61–376	0.79	28.6	24	15
	^b)^*PP* = 2.355*x* + 59.58	117	0.12	0.129	113	31–263	60–271	0.74	27.6	24	15
	^d)^*TURB* = 2.01*x* + 19.19	119	0.47	0.067	66	12–190	19–200	0.89	14.7	22	24
2 Savijoki	^c)^*TP* = 3.31*x* + 63.89	134	0.18	0.250	151	64–400	70–325	0.57	52.0	35	24
	^d)^*TURB* = 2.59*x* - 1.05	134	0.17	0.120	67	4–230	4–203	0.78	25.0	37	22
3 Eurajoki	^c)^*TP* = 3.277*x* + 19.57	103	1.47	0.198	48	10–180	22–153	0.73	12.0	25	16
	^c)^*TP* = 2.944*x* + 22.07	102	0.40	0.226	47	10–120	24–97	0.62	12	25	15
	^c)^*PP* = 3.167*x* + 9.74	97	0.87	0.178	38	7–157	12–139	0.77	10.7	27	19
	^d)^*TURB* = 1.456*x*	102	1.01	0.49	12	2–56	1–37	0.90	4.8	40	28
4 Yläneenjoki	^c)^*TP* = 3.43*x* + 93.76	32	0.40	0.595	137	64–300	94–252	0.52	40.0	29	22
	^c)^*PP* = 3.46*x* + 46.85	32	0.41	0.528	91	39–250	47–206	0.59	35.3	39	15
	^d)^*TURB* = 2.27*x* + 13.25	32	0.36	0.219	42	8–120	13–118	0.78	14.6	35	29

^a)^Two highest P concentrations excluded from the local calibration

^b)^The point/s associated with the highest *D*_*i*_ excluded from the local calibration

^c)^The point/s associated with *D*_*i*_ > 1 excluded from the local calibration

^a,b,c,d)^The excluded points are shown in Fig. A2

### The Aurajoki River

**Fig. 1:**
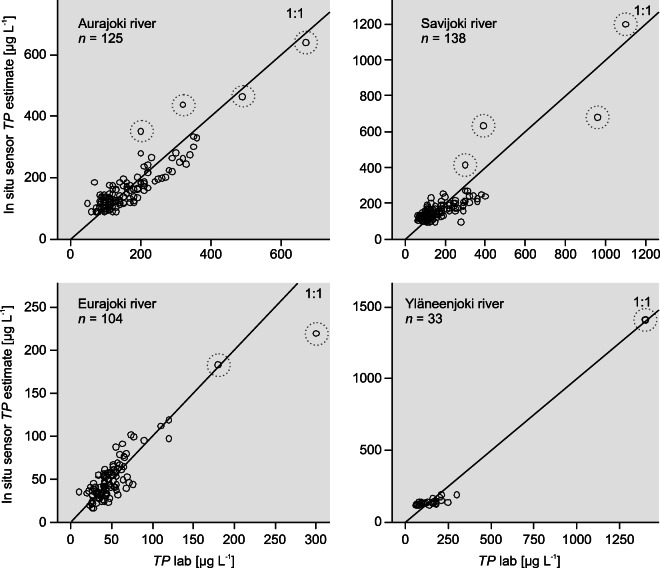
In situ sensor-based total phosphorus (*TP*) concentration estimates versus *TP* determined in the laboratory. The *TP* laboratory data that are considered potentially influential are marked with dashed circles. The *TP* estimates are calculated with the calibration equations given in Table 2

The water sampling represented the discharge range and variation quite well between 2010 and 2013, i.e., during sensor deployment. However, the *TP* concentration range (48–670 μg L^−1^) (Fig. 1) was not as wide as the range (48–1300 μg L^−1^) from 1990 to 2017. However, laboratory data displayed similar regressions between *TP* and turbidity during the two monitoring periods (Fig. A3). There was only a minor difference in the mean ± standard error of the regression slopes (1.34 ± 0.04) of the sensor period data vs. 1.25 ± 0.02 of the long-term data, and the regression intercepts (64.29 ± 3.33 vs. 67.86 ± 2.11) were the same between the two periods. This indicates that the local *TP* calibration equation of the sensor can be applied to an extended concentration range of *TP* up to 1300 μg L^−1^. Overall, the sensor showed a slightly better capability to produce *PP* than *TP* estimates based on the *R*^*2*^, *MSPE* and median *RPD* (Table 3). Consistently, the median *RPD* of laboratory-based *PP* estimates was 13–14%, whereas for *TP*, it was 14–15%, but the *MSPE* values for *PP* were not better than those for *TP* (Table 4). However, there was a large variation between individual chemistry *TP* samples and *TP* estimates (Fig. 1), which was also the case for *PP*.

**Table 4:** Turbidity as an explanatory variable (*x*) for total phosphorus (*TP*) and particulate phosphorus (*PP*) in linear regression models based on water sampling and laboratory analyses. The correlation coefficient (*R*^2^), the root mean squared error (*RMSE*) of the regression model, the model standard percentage error (*MSPE*) and the median relative percentage difference (*RPD*) are also presented

Site and timeframe	Regression model	Max *D*_*i*_	Number of data pairs	*TP* or *PP* range in the laboratory (μg L^−1^)	*TP* or *PP* range estimated (μg L^−1^)	*R*^2^	*RMSE* (μg L^−1^)	*MSPE* (%)	Median RPD (%)
1 Aurajoki, 1990–2017	*TP* = 1.25*x* + 67.86	1.64	726	48–1300	77–1082	0.89	39.7	22	14
	*TP* = 1.22*x* + 70.25	0.54	725	48–1000	79–853	0.88	38.8	21	15
	*PP* = 1.20*x* + 33.41	3.69	622	21–1225	42–1001	0.91	32.4	23	14
	*PP* = 1.13*x* + 38.55	0.33	620	21–776	47–739	0.89	30.3	22	13
2 Savijoki, 1990–2017	*TP* = 1.08*x* + 64.29	5.08	919	29–1100	68–1469	0.83	51.5	31	19
	*TP* = 1.22*x* + 52.63	0.48	916	29–960	56–992	0.85	45.8	28	18
	*PP* = 0.97*x* + 45.70	6.46	41	11–568	51–384	0.51	66.0	58	36
	*PP* = 0.82*x* + 54.87	0.35	39	11–290	59–218	0.34	45.9	48	38
3 Eurajoki, 2009–2014	*TP* = 2.38*x* + 19.45	8.78	108	10–300	24–205	0.76	16.5	33	17
	*TP* = 1.63*x* + 27.51	0.35	106	10–120	31–119	0.61	11.9	25	15
	*PP* = 2.32*x* + 9.73	10.1	102	7–276	15–191	0.82	13.9	35	18
	*PP* = 1.69*x* + 16.40	0.49	100	7–114	20–111	0.74	9.3	25	17
4 Yläneenjoki, 1990–2017	*TP* = 1.16*x* + 70.87	3.87	602	34–1400	73–1347	0.74	38.4	33	23
	*TP* = 1.08*x* + 73.78	0.21	601	34–480	75–506	0.49	38.3	33	23
4 Yläneenjoki, 1990–2015	*PP* = 1.13*x* + 37.12	8.85	540	0–1341	39–1280	0.82	30.3	37	24
	*PP* = 1.73*x* + 0.26	0.32	539	0–431	42–455	0.60	30.0	37	23

### The Savijoki River

The *TP* concentration range of the discrete samples was 64–1100 μg L^˗1^ during sensor deployment (Table 2). However, the majority of the samples only covered the *TP* range of 64–400 μg L^˗1^ (*n* = 135). The visual comparison of *TP* estimates and chemistry *TP* data (Fig. 1) and a large *D*_*i*_ indicate that the calibration equation shown in Table 2 needs to be evaluated against an optional regression curve. *MSPE* (39%) and the median *RPD* (26%) were relatively large. The mean difference between chemistry data and sensor-based *TP* estimates was 47 μg L^˗1^ (*SD* = 44 μg L^˗1^).

The sensor deployment period (2009–2013, *n* = 140) and the long-term laboratory data (1990–2017, *n* = 919) resulted in notably different linear regressions between turbidity and *TP* (Fig. A3), which also indicates that the number of high *TP* concentration (> 400 μg L^˗1^) samples was low in the formation of the calibration equation. Only eight *PP* samples were available for the sensor calibration; thus, a *PP* estimation model was not produced for Savijoki.

Overall, the mean turbidity was similar in Savijoki and Aurajoki (Table 2 and Fig. A1). The sensor-measured turbidity in the Aurajoki main river was better than that in the Savijoki headwater based on *MSPE* and median *RPD*. However, the median difference between laboratory- and sensor-based turbidity was quite similar, with 9 FNU and 10 FNU in Aurajoki and Savijoki, respectively.

### The Eurajoki River

The *TP* concentration of the discrete samples in the local sensor calibration ranged from 10 to 300 μg L^˗1^, but above the concentration 120 μg L^˗1^, only two samples were available (Fig. 1). Note that discrete samples prior to sensor deployment were not available from the Eurajoki sensor location. The median *RPD* values of the *TP* and *PP* estimates were 21% and 22% (Table 2), respectively, whereas the median absolute difference was 9 μg L^−1^ and 8 μg L^−1^ for *TP* and *PP,* respectively. The median *RPD* of 29% for the turbidity estimates was larger than that of the *TP* or *PP*. However, the turbidity level (mean: 13 FNU) was clearly lower in Eurajoki than at Sites 1 and 2. The mean difference between the sensor and laboratory-based turbidity was only 3.7 FNU (*SD* = 3.0 FNU).

### The Yläneenjoki River

The median *RPD* was 30–31% for all water quality constituents, as one potentially influential point with high *D*_*i*_ was within the regression and resulted in high *R*^2^ values (Table 2). The influential sample collected on May 20, 2014, had the highest *TP* concentration of 1400 μg L^−1^ and a turbidity value of 1100 FNU (Fig. 1). The rest of the discrete *TP* sample concentrations only reached 300 μg L^˗1^, and turbidity reached 120 FNU. An analysis of the constituent relationships without the influential sample is needed, and those equations are shown in Table 3.

The long-term laboratory data (1990–2017, *n* = 602) showed a rather similar regression between *TP* and *TURB* than the shorter sensor deployment period laboratory data (*n* = 35) due to the abovementioned sample with a high *TP* concentration (Fig. A3). Exclusion of that point from the long-term *TP* estimation model resulted in the following model: *TP* = 1.08 *TURB*_lab_ + 73.8 (*R*^2^ = 0.49, *n* = 601, *p* < 0.001). However, the exclusion of the point did not introduce a significant change between the regressions (*n* = 602 and *n* = 601) shown in Table 4, since there were no differences in the mean ± standard error of the slopes and the intercepts. The equation (*n* = 601) was formed with data containing seven samples in the *TP* range 300–480 μg L^˗1^, which was not covered by the sensor calibration data. Exclusion of the potentially influential point from the laboratory data covering the period 2013–2015 resulted in the regression equation: *TP* = 0.98 *TURB*_lab_ + 97.5 (*R*^2^ = 0.30, *n* = 34, *p* = 0.001). Thus, the strength of the relationship between *TP* and turbidity is quite low in terms of *R*^*2*^. There was no difference in the mean ± standard error of the regression slopes (0.98 ± 0.27) of the sensor period data vs. 1.08 ± 0.05 of the long-term data, but the regression intercept (97.5 ± 13.4 vs. 73.8 ± 2.3) differed slightly between the two datasets. Hence, extrapolation of the sensor *TP* calibration equation (Table 3) beyond the *TP* concentration of 300 μg L^˗1^ should be made with caution.

### Turbidity as a surrogate for *TP* and *PP* based on discrete water sample data

The correlation of *TP* or *PP* with turbidity was significant (*r*_*s*_ *= 0.38*–0.85*, p* < 0.001) at all sites, but the strength varied. *TP* had a similar correlation with turbidity in Aurajoki (*r*_*s*_ = 0.85, *n* = 726) and Eurajoki (*r*_*s*_ = 0.73, *n* = 108). In addition, the *PP* correlation in Aurajoki (*r*_*s*_ = 0.85, *n* = 622) and Eurajoki (*r*_*s*_ = 0.84, *n* = 102) was similar. In Savijoki, *PP* (*r*_*s*_ = 0.45, *n* = 41) correlated less with turbidity than *TP* (*r*_*s*_ = 0.81, *n* = 919), whereas in Yläneenjoki, the correlation of *PP* (*r*_*s*_ = 0.54, *n* = 540) with turbidity was stronger than that of *TP* (*r*_*s*_ = 0.38, *n* = 602). Thus, in all but one site, *PP* correlated equally or better than *TP* with turbidity. The mean *TP*/*TURB* ratios and standard deviation (*SD*) were 2.6 (*SD* = 1.4) in Aurajoki, 2.5 (*SD* = 3.1) in Savijoki, 5.0 (*SD* = 2.1) in Eurajoki and 5.0 (*SD* = 5.3) in Yläneenjoki. Thus, for the *TP*/*TURB* and *PP/TURB* ratios, the variance was larger in the sub-catchments compared with the larger catchment sites.

Linear regression models were fitted to all long-term discrete water samples, and additional models were formed by excluding points with *D*_*i*_ > 1 (Table 4 and Fig. A3). In terms of *R*^*2*^, the relationship between *PP* and turbidity was better than that between *TP* and turbidity at three sites: Aurajoki, Eurajoki and Yläneenjoki (Table 4). However, according to the *MSPE*s, turbidity is not a better surrogate for *PP* than for *TP.* The median *RPD* of the *TP* estimates ranged from 14 to 23%, whereas for *PP*, the median *RPD* ranged from 13 to 38%. The median *RPD* value of the Savijoki *PP* estimate was high compared with that of other sites.

There was a notable deficiency in the quality of the *TP* or *PP* estimates at the minimum concentrations and at low concentrations when the largest *RPD*s were found. Thus, the lowest P estimates were systematically larger compared with the minimum P chemistry values (Table 4). The constants of the regression models determine the minimum P estimates, and those were always well above the minimum P concentrations based on chemistry analyses. Both under- and overestimation of the maximum concentrations occurred, and the match between discrete and estimated high P concentrations was highly dependent on the slope of the regressions. The difference between the maximum laboratory-determined *TP* concentrations and the estimated *TP* concentrations varied from ˗32 to 34%. The *PP* models estimated the maximum *PP* values with a difference ranging from ˗32 to 6%.

**Fig. 2:**
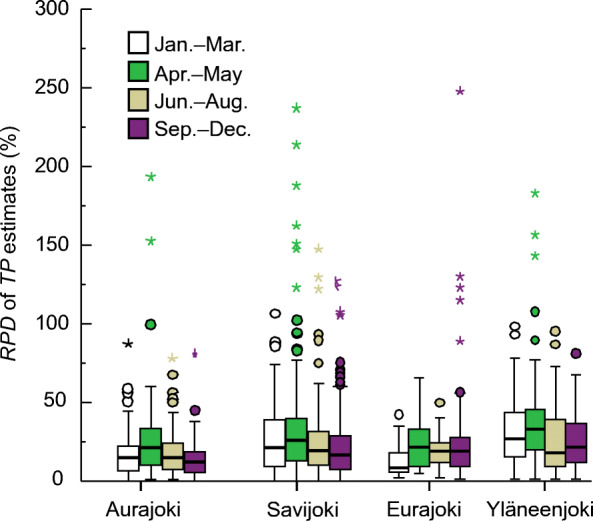
The relative percentage difference (*RPD*) between total phosphorus (*TP*) laboratory concentrations and *TP* estimates produced with linear regression models (see Table 4). The vertical lines within the boxes are the median

We calculated monthly *TP* estimates and recognized seasonal variation in monthly *RPD* values. On a monthly basis, the median *RPD*s of the *TP* model of Aurajoki varied between 8 and 22%. The highest median *RPD*s that appeared in April and May were 22% and 20%, respectively. Similarly, at other sites, the median *RPD* of *TP* estimation models was elevated during the spring months of April and May compared with other seasons (Fig. 2).

### Potentially influential water samples

Visual inspection of the regressions between explanatory raw sensor data and chemistry *TP* and *PP* data indicates some potentially influential points, which if removed from or included in the regression may introduce a difference in the local sensor calibration and generated concentration estimates (Figs. 1 and A2). The hypothesis is that if *D*_*i*_ > 1, it indicates a highly influential point. We evaluated the effect of a few high *TP* and *PP* concentrations as well as points with *D*_*i*_ > 1 in local calibration models.

**Fig. 3:**
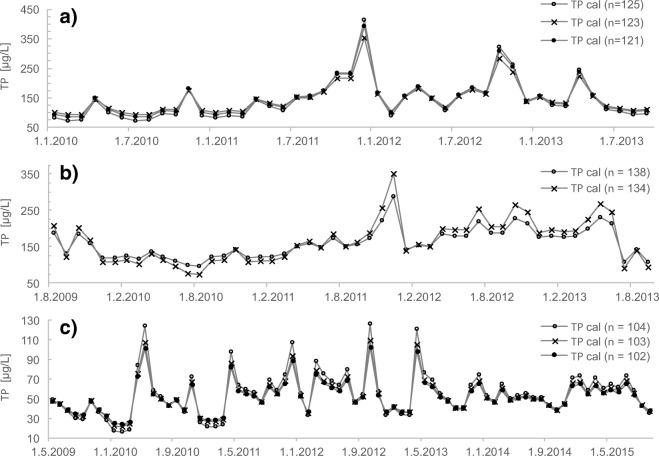
The monthly mean sensor-based calibrated total phosphorus (*TP*) concentrations. The local calibration results are shown with and without potentially influential data for **a** Aurajoki, **b** Savijoki and **c** Eurajoki

The Aurajoki *TP* estimation model had no data with *D*_*i*_ > 1. The removal of the two points with the highest *TP* concentrations had a slight impact on the slope and intercept of the local calibration equation (Tables 2 and 3) and introduced an average difference of 13 μg L^−1^ (11%) in the monthly mean *TP* estimates between the two models (*n* = 125 and *n* = 123) (Fig. 3). However, the removal of the two high *TP* points increased the maximum *D*_*i,*_ and two additional points were removed. This resulted in an average 7% difference only in monthly mean *TP* estimates between the original (*n* = 125) and the optional model (*n* = 121), and the difference was less than 10% during 30 months out of 45 (Fig. 3). The monthly mean *TP* concentration was the highest in December 2011, and the difference between the *TP* estimates was 6–14% (Fig. 3). The model (*n* = 121) had a better *RMSE* compared with the original but is technically applicable only at lower *TP* concentrations. Thus, we recommended applying the original *TP* model for Aurajoki. Exclusion of one point from the local *PP* calibration in Aurajoki resulted in an average 2% effect on monthly *PP* estimates. The effect size was 5–7% when the monthly PP concentration was large (> 270 μg L^−1^). Despite the minor effect of the point on the regression, we suggest removing it from the regression, since the *PP* concentration (95 μg L^−1^) of the point is low and it seems a true outlier based on visual analysis (Fig. A2). However, we decided to keep the two high *PP* values considered potentially influential points in the *PP* calibration. Their exclusion does not change the regression slope when the standard error of the estimate is taken into account or notably improves the error statistic of the local calibration, but they provide information about the *PP* and turbidity relationship at high *PP* concentrations.

**Fig. 4:**
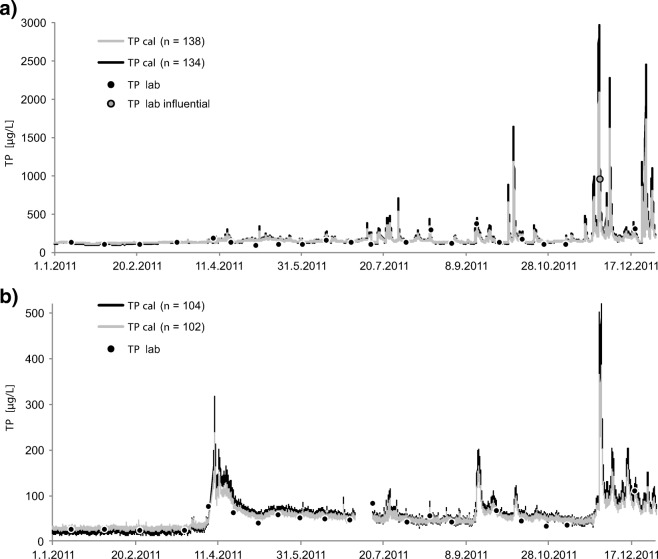
Total phosphorus (*TP*) concentration based on grab samples and sensor estimates with two calibration curves at **a** Savijoki and **b** Eurajoki

In Savijoki, four points were considered potentially influential (Fig. 1), and after removal of those from the local calibration, *D*_*i*_ improved notably (Tables 2 and 3). Two of those samples were collected on the same day, 7 October 2009. The first sample (*TURB* = 1300 FNU, *TP* = 1100 μg L^˗1^) was collected prior to the peak flow of a discharge event, and the second sample (*TURB* = 690 FNU, *TP* = 390 μg L^˗1^) was collected during the falling limb of the flow hydrograph. The two other potentially influential samples were collected in November 2011 and August 2009. A calibration model (*n* = 134) without potentially influential data covered the *TP* concentration range 64–400 μg L^˗1^ very well. The exclusion of potentially influential data improved the median *RDP* value from 26 to 24%. However, this introduced a large difference in estimated *TP* concentrations during high discharge episodes that typically appear during autumn or spring snowmelt (Fig. 4). The difference in the monthly mean *TP* estimates between the models was 20–24% during June–August 2010 and December 2011 (Fig. 4). The maximum effect of the points on the monthly *TP* estimate reached 64 μg L^˗1^ (20%) in December 2011. However, during 70% of the months, the difference was less than 10%. Based on the analysis, the variation in the relationship between turbidity and *TP* is large, and optional calibrations indicate that at high *TP* concentrations, the uncertainty of estimates is quite high.

In Eurajoki, the two discrete samples with the highest *TP* concentrations were considered potentially influential (Fig. 1). The sample with the highest *TP* concentration (300 μg L^−1^) was collected at the time of the rising limb of the hydrograph of a large snowmelt event that lasted for 10 days from 12 to 22 April 2013 and contributed 18% of the annual discharge volume. The mean estimated *TP* concentrations of that snowmelt event decreased 17% from 204 μg L^˗1^ (model *n* = 104; Fig. A4) to 170 μg L^˗1^ (model *n* = 103) by exclusion of that point with the highest *TP* from the local calibration. Removal of the two influential points one by one improved the median *RPD* of the *TP* estimates from 21% (*n* = 104) to 16% (*n* = 103) and to 15% (*n* = 102). The effect of the optional calibrations on monthly mean *TP* estimates reached 24 μg L^˗1^ (19%) (Fig. 3). The mean *TP* concentration was the highest in October 2012, and the related *TP* estimate was 127 μg L^˗1^ (*n* = 104), 109 μg L^˗1^ (*n* = 103) and 102 μg L^˗1^ (*n* = 102). However, in most months, the influential points had a relatively small effect (< 10%) on the mean *TP* estimates (Fig. 3). In the case of *PP*, the removal of one point from the local calibration improved the model as the median *RDP* decreased from 22 to 19% (Tables 2 and 3). However, the new model is strictly only applicable up to a *PP* concentration of 157 μg L^˗1^, which means that *PP* concentration estimates in high flow situations are highly uncertain.

For Yläneenjoki, removal of the *TP* sample with the highest concentration (1400 μg L ^˗1^) (Fig. 1) increased the mean slope of the calibration curve from 1.6 to 3.4, and the median *RDP* improved notably. The effect of influential data removal was similar for the *PP* calibration (Tables 2 and 3). Overall, we can state that more sampling at higher flow rates is needed to improve the sensor calibration.

### Transferability of local sensor calibration between sites

**Fig. 5:**
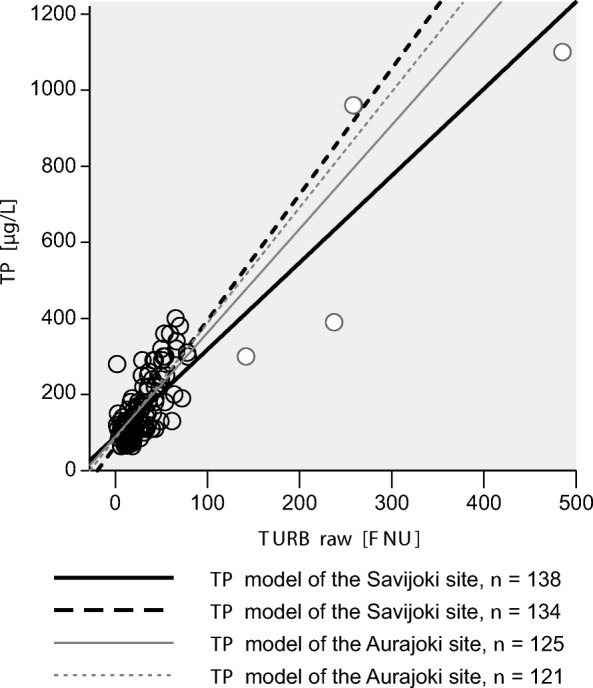
Relationship between raw turbidity (*TURBraw*) sensor values and total phosphorus (*TP*). The dots are *TP* grab samples at Savijoki. White dots indicate potentially influential data. The black lines denote *TP* estimates based on two optional local sensor *TP* calibration models for Savijoki. The grey lines represent *TP* estimates based on the local sensor calibration models of the Aurajoki River, which is the headwater of the Savijoki River. The dashed lines denote *TP* estimates as potentially influential points are excluded from the sensor calibrations

The local *TP* calibration equations of the Aurajoki main river were transferred to its tributary, the Savijoki River (Fig. 5). Naturally, the Savijoki River, which is smaller, has steeper flow slopes and concentration changes than the main river. The transferred equations seem to function as a mean *TP* calibration for the Savijoki sensor, since those fit in between the local sensor calibration equations of the Savijoki site (Fig. 5). There was a small difference only in the mean ± standard error of the regression slopes between the two study sites, of 2.73 ± 0.122 at Aurajoki vs. 2.28 ± 0.107 at Savijoki, whereas the regression intercepts were the same when all data were included in regressions (Table 2). There were no differences in regression slopes between Aurajoki (3.038 ± 0.163) and Savijoki (3.31 ± 0.250) when the potentially influential points were removed from the regressions (Table 3). Hence, the data suggest that calibration of Aurajoki could be transferred to Savijoki.

### The impact of *TDP* on the surrogate relationship between turbidity and *TP* based on grab samples

The applicability of turbidity as a proxy for *TP* may decrease in the case of large temporary variability in the *TDP*/*TP* ratio. The mean *TDP*/*TP* ratio and *SD* were slightly higher in the sub-catchment sites (Savijoki [0.29, *SD* = 0.17] and Yläneenjoki [0.32, *SD* = 0.14]) compared with the larger basins (Aurajoki [0.25, *SD* = 0.12] and Eurajoki [0.26, *SD* = 0.12]). The discrete samples indicated that at high turbidity levels, the *TDP*/*TP* ratio remained low (< 0.2; Fig. A4). Accordingly, the mean and median *TDP*/*TP* ratios were the largest at each site during winter (Jan.–Mar.) when these rivers freeze and the river discharges are typically low. Thus, the *TDP*/*TP* ratio showed some seasonal variation. Despite the mean *TDP*/*TP* ratios being elevated in winter, the *RPD* of the *TP* estimates was not higher during the winter months January–March compared with other seasons (Fig. 2). Seasonally, the lowest mean and median *TDP*/*TP* ratios were found either during summer (for Aurajoki), autumn (for Savijoki), or spring (for Eurajoki and Yläneenjoki).

**Fig. 6:**
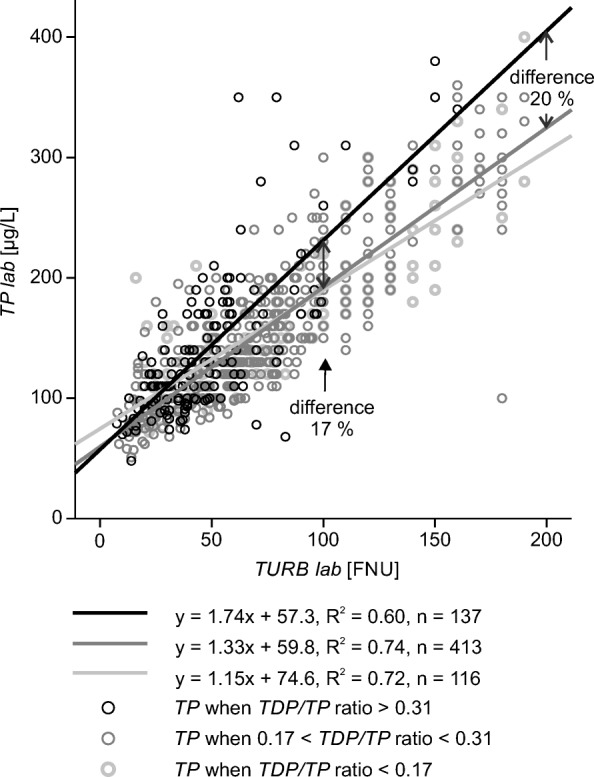
Relationship between turbidity (*TURB*) and total phosphorus (*TP*) based on grab samples collected from the Aurajoki River. The data were divided into three groups based on the ratio between total dissolved phosphorus and total phosphorus (the *TDP*/*TP* ratio). Linear regression models between *TP* and *TURB* are shown for the three groups. The difference between the model-predicted *TP*s increased as turbidity increased. The *p* values of the analyses were less than 0.001

We tested the impact of the varying *TDP*/*TP* ratio on the relationship between *TP* and turbidity at *TP* concentrations lower than 400 μg L^−1^ by splitting the grab sample data of Aurajoki into three subsets according to the level of the *TDP*/*TP* ratio (Fig. 6). The data belonging to the subgroup *TDP*/*TP* > 0.31 represented 20% of the data. The related regression model produced up to 30% higher *TP* concentration estimates than the model based on all data (Table 4). Eighty percent of the observations, at *TP* concentrations lower than 400 μg L^−1^, belonged to the two other groups where the *TDP/TP* ratio was lower and the linear *TP* estimation models differed notably (Fig. 6). Thus, the episodically varying *TDP/TP* ratio has a great impact on the accuracy of some of the *TP* estimates. More precisely, the results suggest that linear models underestimate the actual *TP* concentration episodically when the *TDP/TP* ratio is high.

### *TDP* as an additional explanatory variable in *TP* estimation

The addition of *TDP* as an explanatory variable to the local *TP* calibration of the in situ sensors could potentially improve the *TP* estimation models. Multiple linear *TP* estimation models were built for Sites 1, 3 and 4 (Table A3) since *TDP* data were not available for Savijoki. Analysis indicated the absence of multicollinearity between *TDP* and raw turbidity since the correlation between the predictors was less than 0.7 at all sites. The *R*^2^, *RMSE* and *MSPE* of *TP* estimation improved at all three sites. Median *RPD*s were 12%, 15% and 12% for Sites 1, 3 and 4, respectively. This indicates that the addition of *TDP* to local calibration improved the *TP* estimates notably at the Aurajoki and Yläneenjoki sites, but for Eurajoki, the median *RPD* remained similar.

## Discussion

We calculated *TP* and *PP* concentration estimates with turbidity as a proxy. The differences in the error statistics (*MSPE*, median *RPD*) between the *TP* and *PP* estimates were small. This indicates that turbidity is an equal proxy for both *PP* and *TP* concentrations in rivers draining catchments typified by Vertical Cambisol, Haplic Podzol and Lithic Leptosol soils. The local sensor calibrations resulted in a median *RPD* of 15–24% for *TP* and 15–19% for *PP* at the studied sites. These values were found when the influential samples were removed, which may reduce the applicability of the sensors in producing reliable enough estimates at high P concentrations. Earlier, Christensen et al. (2002) reported median *RPD* ranging from 10 to 17% for *TP* estimates in four Kansas streams. Their results were based on only 1 year of data. It is obvious that our data are more comprehensive, including a wider range of natural water quality variations as well as multiyear sensor and laboratory data. The calculated error statistics in our case indicate a slightly lower performance level of the surrogate equations, potentially because of the larger range of water quality variation described by our more extensive amount of monitoring data. The *MSPE* values of the *TP* surrogate equations for the larger and smaller watersheds were 25% and 29–35%, respectively. These values are similar to the *TP* surrogate equation results presented by Spackman Jones et al. (2011), which showed an *MSPE* of 26–35% for two sites in the Little Bear River in the USA. Overall, sensor raw turbidity functioned better as a proxy for *TP* and *PP* for the two larger rivers compared with their sub-catchments. The *TDP*/*TP* ratio was higher and had a larger variation in the sub-catchment sites. In addition, the *TP*/*TURB* and *PP*/*TURB* ratios had a larger variance in the sub-catchment sites compared with the larger ones. These variations are likely to at least partly explain the better performance of the *TP* and *PP* estimation models for the larger rivers, which is consistent with the findings of Spackman Jones et al. (2011). We assumed that the sensors would provide better estimates for *PP* than *TP* due to light scattering properties of particles carrying *PP*. The relationship between in situ measured turbidity and *PP* was slightly stronger than that between turbidity and *TP*, although *MSPE* indicated varying results for turbidity as a surrogate for *PP* and *TP.*

We used long-term laboratory data parallel to the short-term laboratory data collected during the deployment of the sensors. This proved beneficial in the analysis of the quality and applicability of the local sensor calibrations. We found long-term data (provided by the national surface water quality monitoring programme) useful since the data included additional high flow events associated with high turbidity values and P concentrations, which were rarely captured during sensor deployment despite intentions to collect samples at high flows. Aurajoki’s long-term data included a decent number of samples that covered large turbidity and *TP* ranges. The *TP* concentration estimates based on the long-term and shorter sensor deployment period discrete samples showed similar regression equations for Aurajoki. Thus, the Aurajoki local sensor calibration equation was found to be applicable to a larger P concentration range than the range of the discrete samples used for the sensor calibration.

The median *RPD* of the *TP* estimates, based on long-term discrete samples, was between 14 and 23% (*n* = 106–919) depending on the site considered. These values were on the same level as those for the sensor-estimated *TP* concentrations. We compared individual sample pairs (in situ measurement and discrete water samples) and water quality concentrations produced with different instruments; thus, part of the calculated difference is due to the difference between the samples and analysis methods. The overall performance level of the turbidity sensors with regard to producing high-frequency *TP* and *PP* estimates seems acceptable when keeping in mind that many sources of uncertainties are also involved in water sampling and laboratory analyses. In general, the acceptable measurement uncertainty is ± 20% for turbidity and ± 15% for *TP* and *PP* laboratory data, which are available at lower frequencies, and sampling is often lacking during short-term storm events. The high frequency in situ monitoring provides an extensive amount of data on the turbidity variation, but a linear relationship between turbidity and *TP* or *PP* does not fully explain the temporal variation in turbidity and P ratios that was present in the studied sites. This increases the uncertainty of *TP* and *PP* estimates based on turbidity sensor data. Thus, it is important to be aware of the strengths and limitations of proxy estimations and the applicability of *TP* and *PP* concentration estimates when evaluating catchment dynamics over different time scales.

A major portion of annual nutrient loading from diffuse sources takes place during autumn storms and spring snowmelt storms; thus, capturing P concentrations during these events is essential. Typically, water quality constituents respond in a different manner to flow changes at the rising and falling limbs of the flow hydrograph (Kämäri et al. 2018; Lloyd et al. 2016). Thus, hysteresis may have a notable effect on the local calibration of sensors and may introduce bias into P concentration estimates if turbidity is used as a proxy for P. In Eurajoki, we demonstrated a difference as large as 17% in the mean *TP* concentration estimates of a large snowmelt event based on two optional sensor calibration equations. This finding is consistent with the work of Minaudo et al. (2017), who reported that *TP* concentration estimates during high discharge episodes were subject to considerable uncertainty, dependent on the storm event considered, as they used a nonlinear model to convert turbidity into *TP*. A nonlinear empirical modelling approach with hysteresis effects included between *TP* and turbidity might improve the modelling result but would require intensive grab sampling and preferably use bankside analysers during several storms (Minaudo et al. 2017). At some sites, improvements to the linear surrogate measures could be achieved by employing spring snowmelt as a categorical explanatory variable according to Spackman Jones et al. (2011). Stutter et al. (2017) found differing calibration relationships between turbidity and *PP* at the rising and falling limbs of a storm event hydrograph but also found that the approach requires intensive water sample-based monitoring. Potentially, storm classification and turbidity sensor calibration based on storm type could improve the calibration results, as suggested by Stutter et al. (2017). The storm-type classification approach would require the multi-year intensive sampling of a large number of storms. It would be worth testing different river sizes, especially if such calibrations were transferable across catchments with similar soils and land use. To date, site-specific calibrations have been judged to be non-transferable between catchments (e.g., Stutter et al. 2017). However, our analysis suggested that the local *TP* calibration model is transferable between the main river and its tributary. It would be worth continuing testing if a common calibration curve could be reasonably applied to similar catchments.

The local calibrations shown could be improved by increasing the number of discrete samples collected during high flow events. It is recommended that an automated water sampler be applied since we showed that regular environmental monitoring rarely captures rapid high-concentration storm events. Potentially, separate calibrations for baseflow and stormflows could be developed in the future in case a larger amount of data clearly indicates a nonlinear relationship between turbidity and *TP*.

On a monthly basis, the highest median *RPD* values of the *TP* concentration estimates in the Aurajoki, Savijoki and Yläneenjoki catchments were found in April or May. The *TP*/*TURB* ratios were consistently relatively low in April and May. Accordingly, *PP*/*TURB* ratios were lower than average from April to May at each site, reflecting snowmelt and flow change impacts to some degree. In line with this, Stubblefield et al. (2007) reported a decreased ratio between *TP* and total suspended solids during increased spring flows. In an earlier study, Spackman Jones et al. (2011) found that the *TP*/*TURB* ratio varied between snowmelt and baseflow conditions and reported a categorical variable for spring snowmelt that was significant for *TP* estimates.

The lowest *TP* and *PP* concentrations were systematically overestimated by the sensors. The relatively large intercepts (13–90 μg L^−1^) of the linear *TP* regressions when compared with minimum laboratory concentrations were resulted in an overestimation of the lowest *TP* concentrations. Some earlier study results show relatively large intercepts in regression equations for *TP* estimates (e.g., Christensen et al. 2002), and Spackman Jones et al. (2011) reported a notable amount of *TP* concentration estimates below a detection limit. Thus, we suggest further studies on suitable solutions for handling low concentrations in relation to surrogate use in riverine in situ monitoring. In addition, the range of the *TP*/*TURB* ratio increased considerably during low turbidity conditions and introduced bias between *TP* estimates and discrete *TP* samples. However, the minimum P concentrations appeared during low flows; thus, the bias in low flow P estimates likely does not have a considerable impact on annual P load estimates.

The extensive long-term laboratory data only showed a moderate correlation (*r*_*s*_ = 0.4) between turbidity and *TP* in Yläneenjoki. Seasonal catchment dynamics and land-use practices introduced the most pronounced temporal variation in the *TP*/*TURB* ratio since, from June to the end of October, the mean *TP*/*TURB* ratio (mean = 8.1, *SD* = 6.9) was more than two times higher than the mean of November–May (mean = 3.1, *SD* = 2.7). The Yläneenjoki catchment has more poultry than the other sites, and chicken manure is applied to fields in spring. This may have potentially contributed to the occasionally highly elevated *TP*/*TURB* ratios found during summer and early autumn. Overall, the *TP*/*TURB* ratio was more stable in Aurajoki and Eurajoki than in Savijoki and Yläneenjoki.

The influential samples had a varying impact on the *TP* estimates. During autumnal high flows or spring snowmelt events, the mean *TP* concentrations were notably different, depending on the selected sensor calibration model. Thus, the performance of turbidity sensors in estimating the *TP* concentration is questionable, despite the high measurement frequency, if the temporal variation in the *TP* and turbidity ratio is not captured well enough with local *TP* estimation models at high flow events that contribute a large share of annual nutrient fluxes. A large *D*_*i*_ in regression does not automatically mean exclusion of a point from the regression, but additional evaluation of the point’s influence on the regression slope and intercept is needed.

The median *TDP*/*TP* ratio was the highest at each site during the winter season, but we were not able to generate a seasonally different relationship between turbidity and *TP* since the variation in the *TDP*/*TP* ratio was also large within seasons. Thus, short-term variation in the *TDP*/*TP* ratio caused inaccuracy in the *TP* estimates generated with the turbidity surrogate. This result is in line with that presented by Lannergård et al. (2019), who found that season was not a significant factor in the relationship between high-frequency turbidity and *TP*. The addition of discharge as an additional explanatory variable for the P estimation model might improve *TP* estimation at some sites. Minaudo et al. (2017) used discharge as a proxy of reactive P.

Variation in the *TP*/*TURB* and *TDP*/*TP* ratios caused inaccuracy to the performance of the linear P regression models, which is partly related to hydrological conditions. These observed variations likely also reflected local land-use practices and temporal shifts in the transportation pathways of *TP* compared with turbid matter; in addition, these variations likely reflected the origin of the operational P fractions observed in river water. For example, the organic matter content of turbid matter impacts P concentrations. P-rich organic matter and fine sediments typically accumulate within the reach during low flows and have been reported to mobilise during high flow conditions (Evans et al. 2004). The slope of the linear regression model between laboratory-determined *TP* and turbidity was the highest as the *TDP/TP* ratio was large in the Aurajoki site. The result indicates that periodically, part of P was transported in a form that does not scatter light and was not detectable with the nephelometric method. We observed that the addition of *TDP* to the *TP* regression model as an additional explanatory variable improved the regression. Schilling et al. (2017) concluded that in some rivers, the inclusion of orthophosphorus in the *TP* regression model was less important, but at other sites, it was critically important. However, it is not possible to measure *TDP* continuously with a UV-Vis probe, which hampers the usability of *TDP* in the conversion of in situ sensor data into *TP* estimates. Of the future developments, perhaps, the best approach would be to have a high-frequency P device that can directly measure the P concentration of water in the field. Until then, however, there is still a need to investigate the *TP/TURB* ratio in waters with varying *TDP* concentrations. Further studies should focus on stormflow events when the nutrient load is at its highest.

## Conclusions

This study analyses how well *TP* and *PP* concentrations can be captured with in situ high-frequency UV-Vis turbidity sensors in rivers draining clay-dominated catchments where *TP* and *PP* correlate with turbidity to varying degrees. We found that turbidity is an equally good proxy for *PP* and *TP*. Thus, the error statistics of *PP* and *TP* estimates were quite similar. The median difference between the discrete P samples and the high-frequency P estimates ranged from 15 to 24% depending on the site considered. We presented the challenges that are faced in the use of sensor turbidity as a proxy for P in practice. Local sensor calibration is a crucial point in cases where surrogates are used. Preferably, a large number of discrete samples covering the entire range and variation in turbidity and P concentration are needed, but in practice, high flow events are rarely sampled without an automated sampler. In addition, linear regression and turbidity as a surrogate for P may introduce a large bias, even in annual P load estimates when large flow events comprise a considerable share of annual water yield. We quantified that optional sensor calibrations (i.e., with and without influential samples) introduced a 17% difference into estimated mean *TP* concentrations during a snowmelt storm. However, for most months, influential samples accounted for less than a 10% difference in the monthly mean *TP* concentration estimates between two optional calibrations. To overcome the lack of discrete samples with high P concentrations during sensor deployment, we additionally analysed the relationship between turbidity and P based on long-term discrete sampling (1990–2017). We showed that the analyses of long-term data in parallel with short-term in situ monitoring period data are useful since they may, on the one hand, show an increased applicability range of sensor calibrations or, on the other hand, suggest that more sampling is needed for sensor calibration. This approach is recommended as a common practice in local in situ sensor calibration.

The data suggested that linear regressions between *TP* or *PP* and turbidity do not fully describe actual *TP* and *PP* concentrations in the rivers. A large seasonal variation in the relationship between turbidity and *P* values, the hysteresis effect and the variation in the *TDP/TP* ratio decreased the performance of the regression models. The *RPD* between discrete *TP* and *PP* samples and estimated samples was large, especially at low concentrations, since the constants of the calibration equations that determine the minimum concentration of P estimates were larger than the minimum laboratory-based P concentrations. The removal of influential observations improved the overall performance of the regression models, but simultaneously, the representativeness of calibrations at the highest *TP* or *PP* concentrations became uncertain. A large share of the annual P load takes place during relatively short-term runoff events during snowmelt and autumn; thus, it is crucial to accurately estimate the high P concentrations during these events. In the case where the regression model does not adequately describe the high concentrations during high flow events, the uncertainty of annual load estimates may become unacceptable.

We recommend analysing the correlation and regressions of turbidity with P based on intensive laboratory analyses before investing in a turbidity sensor for the purpose of monitoring the P concentration in rivers. More research is needed to define better and cost-effective surrogate calibration methods that could be applied in national water quality monitoring programmes. Research-based discussion on the acceptable uncertainty of event-scale estimates, annual nutrient concentration estimates or load estimates is also encouraged.

## Electronic supplementary material

ESM 1 (PDF 712 kb)
