# Supplementary material for: High-frequency measured turbidity as a surrogate for phosphorus in boreal zone rivers: appropriate options and critical situations
Source: Environ Monit Assess. 2020 May 15;192(6):366. doi: 10.1007/s10661-020-08335-w (PMC7228995; doi:10.1007/s10661-020-08335-w)
Supplement: Supplementary file 1 — (PDF 712 kb) [file 10661_2020_8335_MOESM1_ESM.pdf]

ELECTRONIC SUPPLEMENTARY MATERIAL

High-frequency measured turbidity as a surrogate for phosphorus in boreal zone rivers: Appropriate options and critical situations

Maria Kämäri[1], Marjo Tarvainen[2], Niina Kotamäki[3], Sirkka Tattari[1]

[1]Finnish Environment Institute, Latokartanonkaari 11, FI-00790 Helsinki, Finland

[2]Centre for Economic Development, Transport and the Environment for Southwest Finland, P.O. Box 236, FI-20101 Turku, Finland

[3]Finnish Environment Institute, Survontie 9A, FI-40500 Jyväskylä, Finland

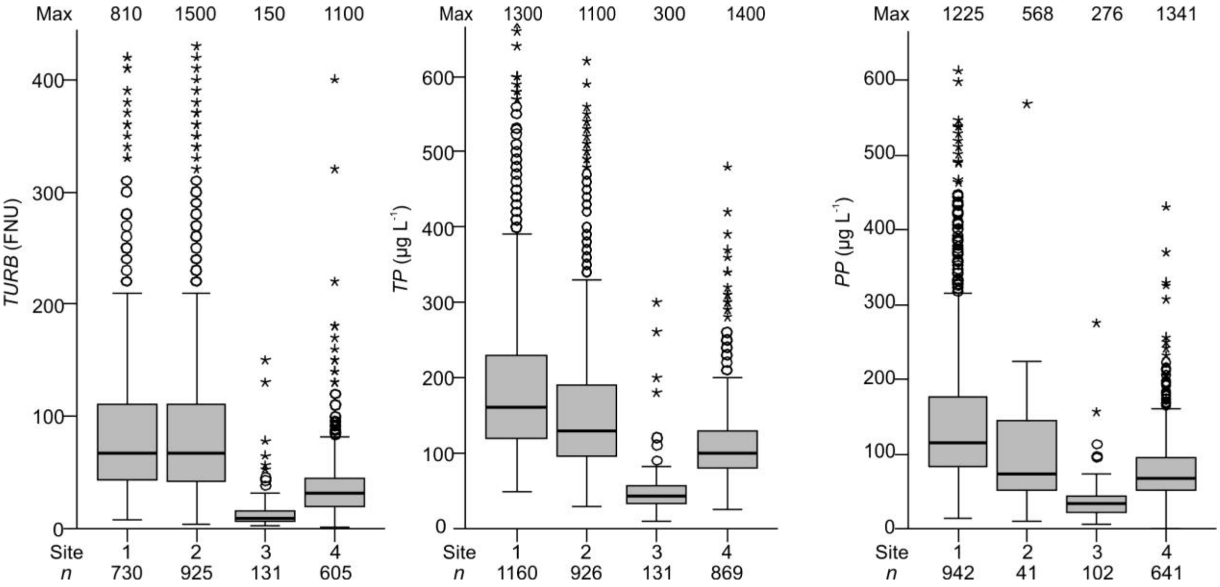

Fig. A1 The median, lower and upper quartiles; the minimum values, outliers and extreme outliers of turbidity (*TURB*); and the total phosphorus (*TP*) and particulate phosphorus (*PP*) concentrations from 1990–2017 at the following monitoring sites: Aurajoki (Site 1), Savijoki (Site 2), Eurajoki (Site 3) and Yläneenjoki (Site 4). The number of samples is denoted by *n*. The number of *PP* samples for Savijoki was only 41, most of which were collected between December 2015 and December 2017 (*n* = 33), and the rest were collected in 2010. In the case of Yläneenjoki, the *PP* data were available until December 2015.

Table A1 Phosphorus (P) classification

**Classification, Haygarth and Sharpley (2000)**	**Classification, this paper**
TP (unf), total P in raw, unfiltered sample (TP)	*TP*, total phosphorus
TP (< 0.45), total dissolved P (TDP)	*TDP*, total dissolved phosphorus (< 0.40)
TP (> 0.45) = TP (unf) -TP (< 0.45), particulate P (PP)	*PP*, particulate phosphorus (>0.40)

Table A2 Goodness-of-fit statistics

**Definition**	**Equation**
Correlation coefficient	$R^2 = 1 - \frac{SS_{Err}}{SS_{Tot}} = \frac{\sum y_i^2 - \beta_0 \sum yi - \beta_1 \sum x_i y_i}{\sum y_i^2 - (\sum y_i)^2 n}$
The root mean squared error (*RMSE*)/standard error of the estimate	$RMSE = \sqrt{\frac{1}{N-k}\sum_{i=1}^{N}(y_i - x_i)^2}$
Model standard percentage error (*MSPE*)/relative *RMSE*	$MSPE = \pm \frac{RMSE}{\bar{x}} \times 100$
Relative percentage difference (*RPD*)	$RPD = (\lvert y - x \rvert / x) \times 100$

Here, *N* is the total number of data pairs, *i* is the sample number, *k* is the degrees of freedom associated with the source of variance (*k* = 1 in cases when the intercept is included in the regression model), *y* is the estimated value and *x* is the actual value i.e. laboratory analysed value. The estimated value is the locally calibrated sensor estimate or a *TP* or *PP* estimate based on the linear relationship between laboratory-determined turbidity and *TP* or *PP*. The least uncertain estimates are those returning the lowest *RMSE*. The *RMSE* values are used to assess the variance between observed and estimated values.

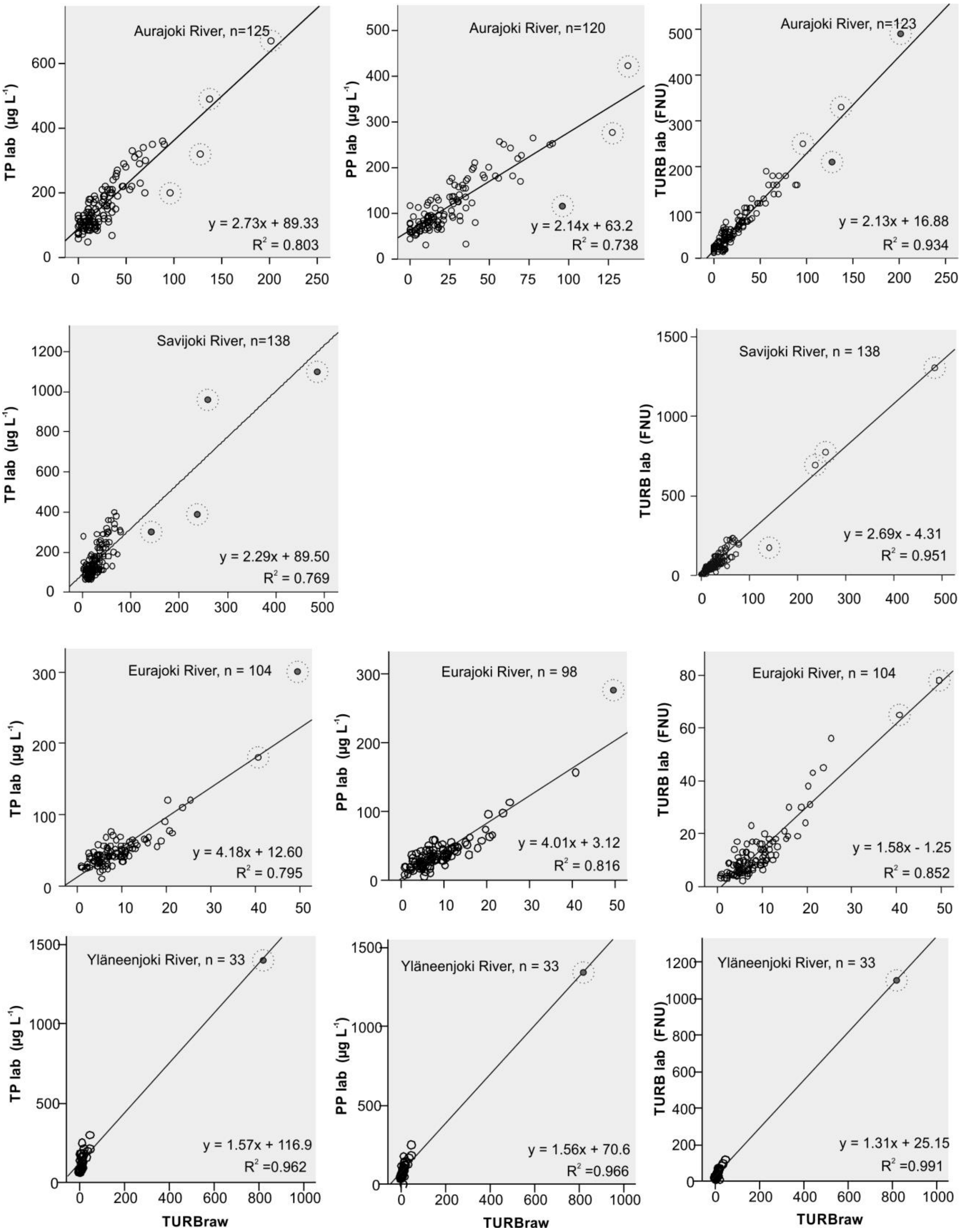

Fig. A2 Regression between explanatory sensor raw turbidity (*TURBraw*) and response variables from discrete water sample data, i.e., total phosphorus (*TP*), particulate phosphorus (*PP*) and turbidity (*TURB*). Dashed circles denote potentially influential *TP* and *PP* data. Dots with grey fill denote points with Cook's distance larger than one.

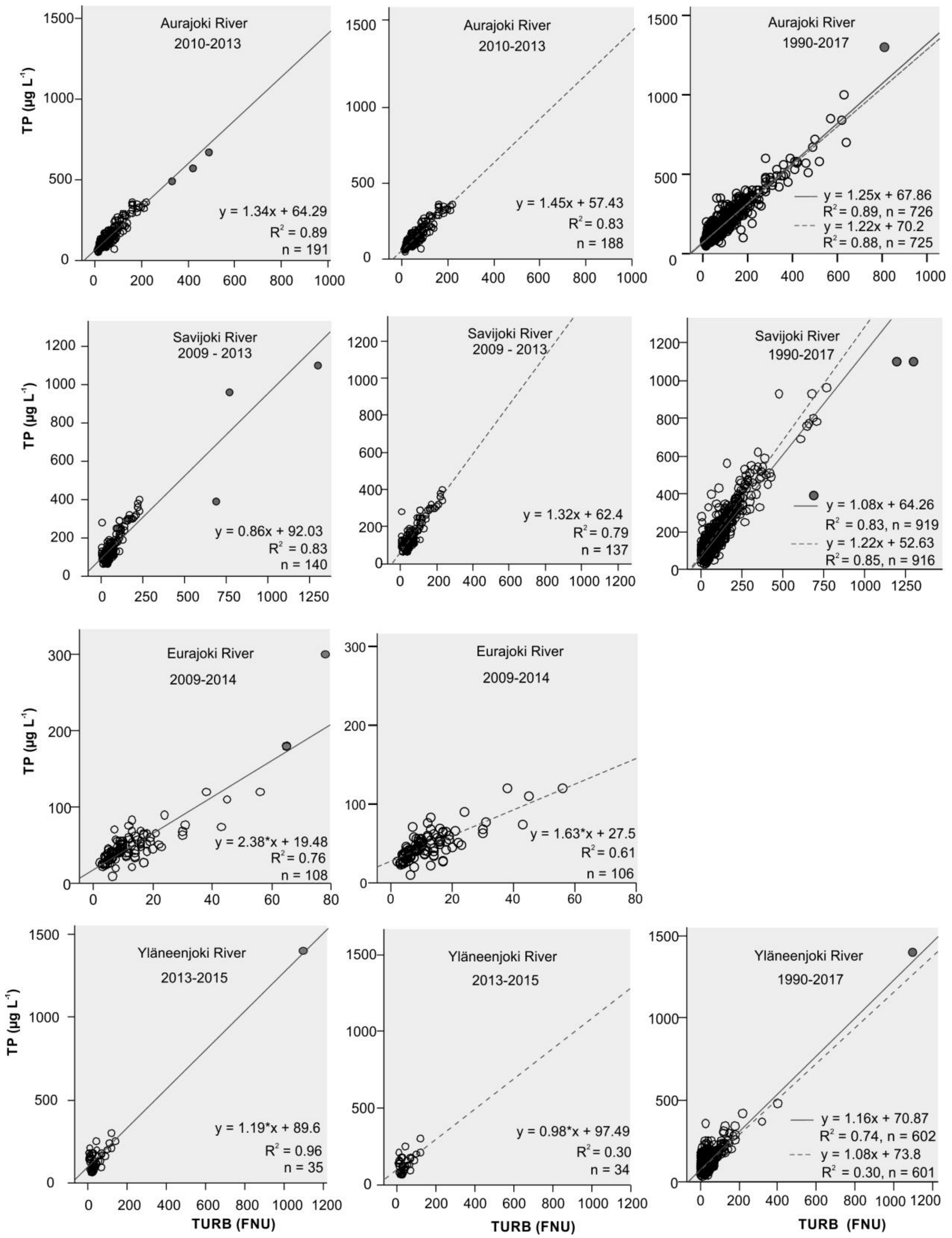

Fig. A3 Turbidity (*TURB*) laboratory samples as a proxy for total phosphorus (*TP*) in linear regression. The panes on the left side represent the data of in situ monitoring periods, and the panes in the middle represent the in situ sensor period model without potentially influential points. On the right are long-term observations and related models with and without influential data. Dashed lines are *TP* estimates without potentially influential data.

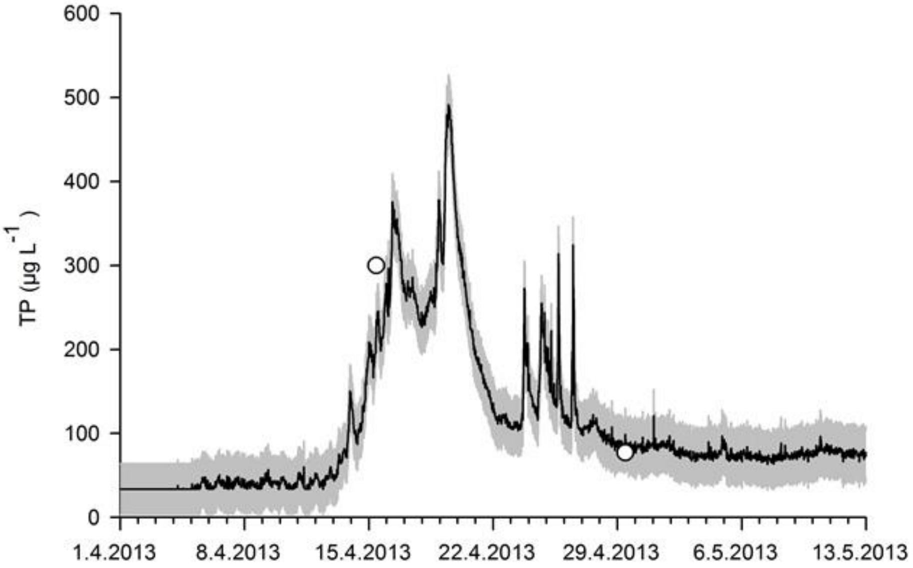

Fig. A4 Estimated total phosphorus (*TP*) (*n* = 104) and the 95% confidence limits during a snowmelt event for Eurajoki. The white circles denote discrete water sample data.

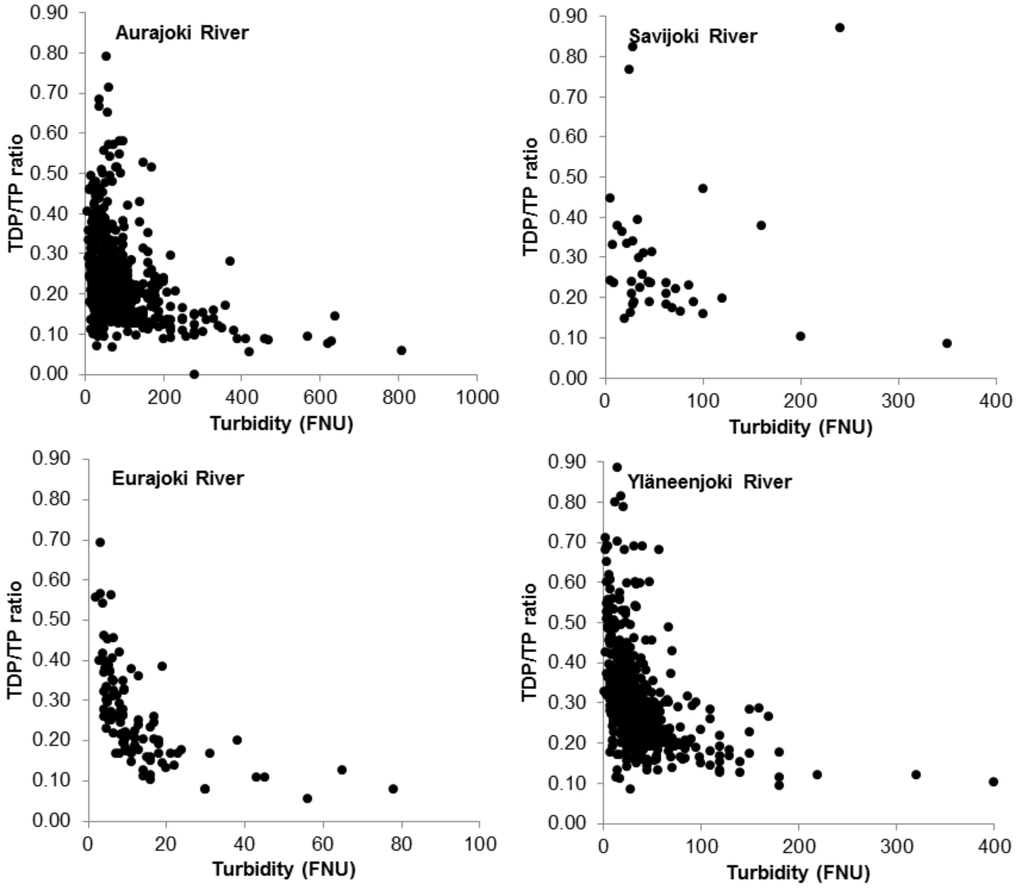

Fig. A5 The total dissolved phosphorus (*TDP*) and total phosphorus (*TP*) ratio versus laboratory values for turbidity

Table A3 Determined regression models to convert in situ sensor raw turbidity values (*x*) into calibrated total phosphorus (*TP*). The regression equations are formed for Aurajoki (Site 1), Eurajoki (Site 3) and Yläneenjoki (Site 4). Total dissolved phosphorus (*TDP*) is used as an additional explanatory variable. Number of data points (n), the maximum Cook´s distance (D_i), mean *TP* concentration of the grab samples, standardized beta coefficients of independent variables, coefficient of determination (R^2), root mean square error (*RMSE*), model standard percentage error (*MSPE*) and median relative percentage difference (*RPD*) are also presented. The standardized beta coefficient of independent variables is used to compare the strength of the effect of raw turbidity and *TDP* to the *TP* estimates. *p*-value is less than 0.001 in all regressions

Site	Regression model	n	Max D_i	*TP* mean (µg/L)	Standardized beta coefficient for *x*	Standardized beta coefficient for *TDP*	R^2	*RMSE* (µg/L)	*MSPE* (%)	Median *RDP* (%)
1	$TP = 1.95x + 1.40TDP + 52.58$	120	0.76	156	0.63	0.39	0.83	31.4	20	12
2	no *TDP* data available	-	-	-	-	-	-	-	-	-
3	$TP = 3.14x + 1.27TDP + 6.92$	97	0.59	49	0.83	0.22	0.79	10.7	21	15
4	$TP = 3.45x + 1.42TDP + 35.43$	30	0.33	138	0.73	0.54	0.81	25.9	19	12
